# Specialized ribosomes: integrating new insights and current challenges

**DOI:** 10.1098/rstb.2023.0377

**Published:** 2025-03-06

**Authors:** Alan J. S. Beavan, Veronica Thuburn, Bulat Fatkhullin, Joanne Cunningham, Tayah S. Hopes, Ella Dimascio, Tessa Chan, Nan Zhao, Karl Norris, Chalmers Chau, Elton J. R. Vasconcelos, Alison Wood, Adrian Whitehouse, Paolo Actis, Brendan Davies, Juan Fontana, Mary J. O'Connell, Emma Thomson, Julie L. Aspden

**Affiliations:** ^1^ Computational and Molecular Evolutionary Biology Group, School of Life Sciences, Faculty of Medicine and Health Sciences, University of Nottingham NG7 2RD, UK; ^2^ School of Biosciences, Faculty of Science, University of Sheffield, Sheffield S10 2TN, UK; ^3^ Faculty of Biological Sciences, University of Leeds, Leeds LS2 9JT, UK; ^4^ Astbury Centre for Structural Molecular Biology, University of Leeds, Leeds LS2 9JT, UK; ^5^ LeedsOmics, University of Leeds, Leeds LS2 9JT, UK; ^6^ School of Electronic and Electrical Engineering, University of Leeds, Leeds LS2 9JT, UK; ^7^ Bragg Centre for Materials Research, University of Leeds, Leeds LS2 9JT, UK

**Keywords:** ribosome, translation regulation, protein synthesis, specialized

## Abstract

Variation in the composition of different ribosomes, termed ribosome heterogeneity, is a now well established phenomenon. However, the functional implications of this heterogeneity on the regulation of protein synthesis are only now beginning to be revealed. While there are numerous examples of heterogeneous ribosomes, there are comparatively few bona fide specialized ribosomes described. Specialization requires that compositionally distinct ribosomes, through their subtly altered structure, have a functional consequence to the translational output. Even for those examples of ribosome specialization that have been characterized, the precise mechanistic details of how changes in protein and rRNA composition enable the ribosome to regulate translation are still missing. Here, we suggest looking at the evolution of specialization across the tree of life may help reveal central principles of translation regulation. We consider functional and structural studies that have provided insight into the potential mechanisms through which ribosome heterogeneity could affect translation, including through mRNA and open reading frame selectivity, elongation dynamics and post-translational folding. Further, we highlight some of the challenges that must be addressed to show specialization and review the contribution of various models. Several studies are discussed, including recent studies that show how structural insight is starting to shed light on the molecular details of specialization. Finally, we discuss the future of ribosome specialization studies, where advances in technology will likely enable the next wave of research questions. Recent work has helped provide a more comprehensive understanding of how ribosome heterogeneity affects translational control.

This article is part of the discussion meeting issue ‘Ribosome diversity and its impact on protein synthesis, development and disease’.

## 1. Introduction

The translation of mRNA into proteins by the ribosome is a tightly controlled step in gene expression. However, until recently the ribosome itself has been seen as a passive player in this regulatory process. With the discovery of compositionally distinct ribosomes, a debate has now arisen as to whether such heterogeneous ribosomes can display specialized function and provide additional layers of regulation to translation, ultimately influencing the translatome of a cell. ‘Heterogeneous’ and ‘specialized’ classifications are not synonymous, i.e. heterogeneous ribosome composition does not necessarily equate to specialization of function [1]. Here we explore the major challenges and questions in the field of ribosome heterogeneity and specialization, and critically evaluate current evidence.

The ribosome is a multi-subunit complex normally composed of approximately 80 ribosomal proteins (RPs) and four rRNAs (five in *Drosophila melanogaster*). One of the first indications that ribosomes could be heterogeneous was reported in the 1980s, where 5S rRNA variants were identified in *Xenopus* [2]. However, whether these early-identified variant forms of rRNA were actually incorporated into ribosomes, and whether this had any effect on their function, remained unclear. Recent innovations in mass spectrometry, Ribo-Seq and direct-RNA sequencing approaches have enabled numerous examples of both ribosomal protein and rRNA heterogeneity to be described in diverse model organisms, tissues and cell types under different physiological conditions (e.g. developmental and stress) [3–6]. Heterogeneity in protein composition of the ribosome can arise through the incorporation of alternative RP paralogues, differential post-translational modification and changes in the stoichiometry [3,4,7–11]. Similarly, the rRNA component of the ribosome has been shown to vary at the primary sequence level and through changes in modification pattern [5,12–15]. Compositional variation in ribosome-associated proteins has also been reported [16].

The apparent widespread nature of heterogeneity, in different organisms and across different tissues, under various physiological conditions (e.g. cellular stress) and diseases states, has led to a general acceptance that ribosome composition has a greater plasticity than previously appreciated. However, while numerous examples of heterogeneity have been described [3–5,17,18], many outstanding questions remain regarding the nature of heterogeneous components. These include questions on the scale of heterogeneity, whether heterogeneous ribosomes are conserved across species, if different heterogeneous components combine with one another, and how heterogeneity affects the interaction of ribosome-associated proteins.

In its most basic form, specialization is where compositionally distinct subsets of ribosomes within a population regulate translation in a different manner from other ribosomes and alters the translational output. Further, while there are numerous examples of heterogeneous ribosomes seen in diseases, such as oncoribosomes [19–22], which dysregulate translation, a distinction should be made from specialized ribosomes that function under homeostatic conditions.

While there are numerous examples of heterogeneous ribosomes, there are comparatively few bona fide specialized ribosomes described. The best characterized examples include eS26-containing ribosomes in yeast [6,23], eL38 in mouse [24] and BUD23 in Kaposi sarcoma-associated herpesvirus (KSHV) infection [18]. Key pieces of evidence that support such examples includes the characterization of different ribosome populations that occur without exogenous disruption by RNAi/CRISPR, etc. Populations might be induced by stresses (e.g. starvation in yeast, or viral infection) or during development (eL38). This altered population needs to differentially regulate translation of a specific pool of mRNAs or open reading frames (ORFs) compared with other ribosomes. For example, lytic KSHV infection results in 40S ribosomes with increased G1823 methylation, which changes the level of KSHV upstream ORF (uORF) and main ORF translation [18].

Even for the best examples of specialization, where detailed structural models are available, our mechanistic understanding of how different forms of heterogeneity give rise to specialization is limited. Part of the difficulty in assigning specialization status is the challenge in defining what a specialized ribosome is and what properties and features it should exhibit.

**Figure 1.**
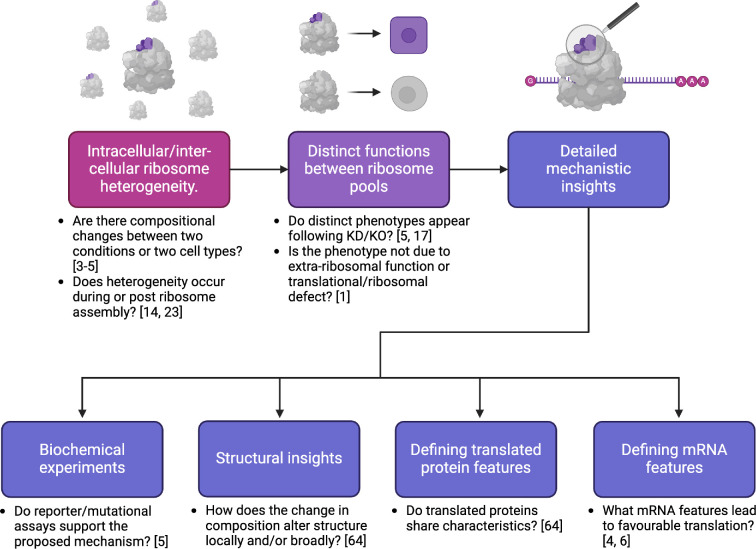
Best practice to identify and characterize specialized ribosomes. Flow-chart depicting questions to answer to comprehensively establish ribosomes with specialized function, from the initial discovery of heterogeneity to elucidating the detailed mechanism. The latter is split into four parts. References in this diagram are considered to be gold standard examples of where the question has been extensively answered. Knockout (KO), Knockdown (KD).

As natural philosophers our biological definitions are conceptual frameworks to aid our understanding and communication of complex biological phenomena. While a ‘definition’ is not an absolute truth and may be subject to refinement and adaptation, an operational-level definition is critical for rapid progress in empirical studies. Of critical importance are the key measurements and observations necessary for classification of ‘specialization’ (figure 1). We discuss the desiderata for ‘ribosome specialization’ and the challenges in identifying and understanding mechanisms of action, in addition to critically evaluating experimental methodologies used to determine specialization.

## 2. Defining specialization in the context of evolution

Putative examples of specialization span eukaryotes, from plants and animals, to fungi and single-celled ‘protists’. The most parsimonious explanation for the distribution is that at least some elements of specialization existed in the last eukaryotic common ancestor (LECA) [4,7,25,26]. Addressing the deeply fundamental question of the extent to which modes of specialization are ancestral or derived is central to constructing our definition, providing us with further insight into whether there are genuine categories of specialization in nature and whether our definition(s) correspond to these ‘natural kinds’. Determining the natural kinds of specialized ribosomes helps us to crystallize our definition of specialization and understand the context in which specialized ribosomes emerged. That is, if a feature of specialization was in the LECA, this could be considered a core mode of specialization, whereas more derived modes of specialization could be considered supplementary. These putative categorizations do not mean that ancestral/core modes are more likely to be biologically significant than derived/supplementary modes. Indeed, both are likely to have facilitated the evolution of gene regulation through translational control. Instead, these terms are designed to help us understand the landscape of specialization, i.e. they contribute to a working nominal definition. Through understanding the context of emergence, we stand to gain refinement of our operational definition, and greater insight into which model organisms can be used most effectively to understand the general principles of ribosome specialization.

Modes of specialization implicated include paralogue inclusion (ancestral and derived cases), RNA methylation and post-translational modification of ribosomal proteins [18,20,27]. The mode of specialization through alternative paralogue inclusion is evident across eukaryotes, but often the specific paralogues involved have emerged independently in multiple lineages, such as the independent evolution of close paralogues to eL22 in insects, mammals and fungi [3]. In this case, although we observe specialization through duplications of the same gene, the resultant paralogues are considered a derived form of specialization rather than ancestral, i.e. the evolutionary process is conserved but the instance of specialization has evolved convergently. To be considered an ancestral form of specialization, for the animal clade for example, the duplication event that gave rise to the paralogous pair of genes would need to have occurred prior to the diversification of animals. Therefore, to truly understand specialization as ancestral or derived requires the reconstruction of phylogenetic trees with precise resolution of gene duplication events. When the extent to which instances of specialization are ancestral or derived is understood it will help to reveal the ecological role that specialized ribosomes have played during macroevolution. For example, we may ask how specialized ribosomes are tolerated and retained through genetic drift, or, as philosophically favoured in [28]—selectively advantageous (e.g. facilitating different cell types to express their genes differently). Further, retention of gene duplicates does not necessarily equate to specialization, neither does it mean functional conservation or divergence, even when both paralogous copies are retained in the long term. For example, each paralogue may be highly conserved at the sequence level but may be mutually exclusively expressed in different tissues owing to, e.g. *cis* regulatory changes; if the mRNAs translated by the paralogues are not distinct, such cases would not considered specialized [29]. How best to answer the question of how conserved the modes and instances of ribosome specialization are throughout eukaryotes will vary. In many cases, sophisticated phylogenetic methods will allow us to identify and place, for example, gene duplication events that have led to divergent ribosomal protein paralogues involved in putative specialized ribosomes, and, as previously outlined, results from such analyses generate important hypotheses, ideally to be tested experimentally [28]. For other modes, such as specialization through rRNA methylation, a more creative approach is likely to be required because currently the modification of rRNA has to be detected experimentally rather than through comparative genomics; additionally the isolation of ribosome populations based on their rRNA modification state has not yet been achieved.

## 3. Different scales of ribosome heterogeneity

Ribosome heterogeneity refers to the presence of at least two different ribosome compositions within a ‘population’. Eukaryotic ribosome populations have been seen within individual cells, as well as more broadly in single cell or tissue types [2–13]. While numerous examples of ribosome heterogeneity have been identified across eukaryotes, a relatively small proportion have been described as ‘specialized’. This may be due to functional redundancy between compositionally distinct ribosomes in a population, but it could also result from a lack of knowledge about which specific cellular conditions induce a ‘specialized’ response. Recognizing patterns of ribosome heterogeneity in various contexts will likely contribute to understanding how functionality and specialization can arise.

**Figure 2.**
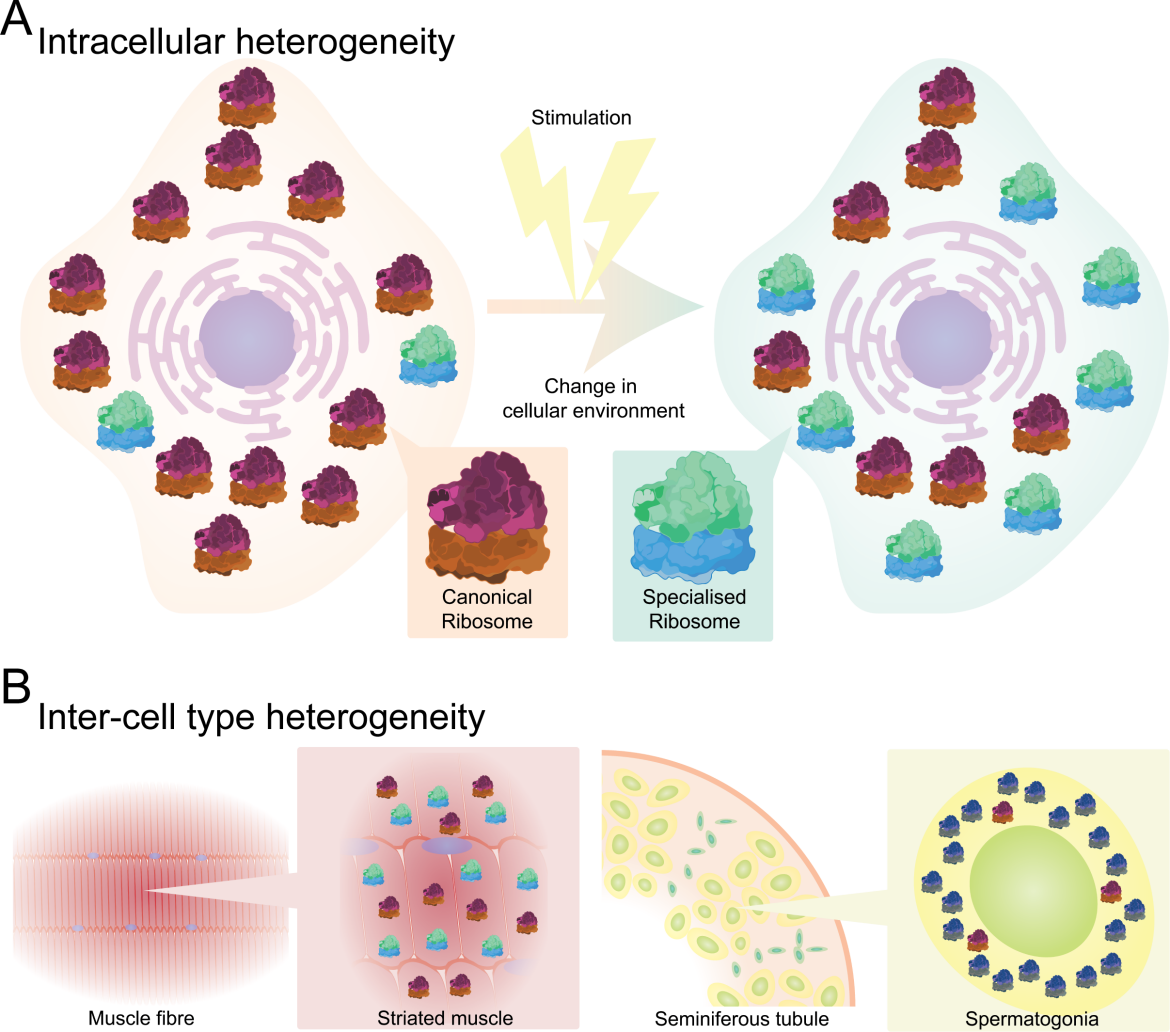
Levels of ribosome heterogeneity. (A) Intracellular heterogeneity. Changes to a cell’s environment can affect the ratio between canonical (pink) and compositionally distinct (green) ribosomes. (B) Inter-cell type heterogeneity. Different heterogeneous ribosomes can be enriched in a tissue-specific manner, such as uL3L ribosomes (green) in striated muscle and eL39L ribosomes (blue) in the male germ cell.

Owing to difficulties isolating a representative sample of an intracellular ribosome population, it is hard to state with certainty that this population is homogeneous. Similarly, the small quantity of a ribosomal population from a single cell makes intracellular heterogeneous populations difficult both to identify and to dismiss. Therefore, intracellular ribosome heterogeneity refers to the ratio between canonical and heterogeneous ribosomes in a single cell or—more frequently—a single cell type where conditions are identical, and how this ratio changes in response to changes in the cell environment (figure 2A). Cases of ribosome specialization linked to intracellular heterogeneity have been observed throughout the eukaryotic domain in response to various physiological cues and cellular stressors including viral infection, abiotic stressors and ageing [18,30,31], as well as between subcellular pools of ribosomes [16].

Two key examples of intracellular heterogeneity that have been characterized involve differential rRNA methylation for protection against oxidative stress. In the microalga *Chlamydomonas reinhardtii*, increased UV intensity causes oxidative stress as well as increased levels of the 18S rRNA methyltransferase Bud23. This is associated with higher levels of the antioxidant pigment lutein and optimizes growth of the organism [30]. While effects of UV on CrBud23 were observed relatively quickly, changes in the synthesis and activity of NSUN5—another rRNA methyltransferase found across eukaryotes—happen on a greater timescale. Transcriptional profiling has indicated that NSUN5 levels were lower in chronologically aged *Saccharomyces cerevisiae* compared with their younger counterparts. Following the production of NSUN5 knockout strains to act as ageing models, translatome characterization before and after induction of oxidative stress has revealed that oxidative stress response genes, which were only upregulated following hydrogen peroxide treatment in wild-type (WT) lines, were upregulated prior to treatment in knockout lines. Therefore, it has been proposed that the absence of NSUN5-mediated rRNA methylation is linked to pre-emptive upregulation of protective stress response genes that improve lifespan in old age [31].

Intracellular heterogeneity presents a yet-unanswered question about the dynamic nature of the canonical : heterogeneous ribosome ratio in a population, and whether this contributes to the transition from a heterogeneous population to a specialized one. Specifically, it is not known whether there is a threshold above which altered translation by non-canonical ribosomes becomes functional, or whether ‘functionality’ itself—the adaptation of a cell to the change in its environment through specific translation—is proportional to the canonical : heterogeneous ribosome ratio in the population.

As in single-cell populations, inter-cellular type heterogeneity can be present in multi-cell-type populations, though their heterogeneity is often more complex. The canonical : heterogeneous ribosome ratio can also differ between cell or tissue types [3,5,32], e.g. mammalian uL3L-containing ribosomes, which are enriched in striated muscle compared with canonical uL3-containing ribosomes but observed at low levels in other tissue types [33,34]. Additionally, different heterogeneous ribosomes have been observed in tissue types with distinct cellular conditions, such as increased energy requirements in striated muscle, or enrichment of eL10L- and eL39L-ribosomes in the male germ cell, whose knockdown reduces fertility (figure 2B) [11,33,34]. It should be noted that both eL10 and eL39 are X-chromosome-encoded so their paralogues likely play a compensatory role during meiosis-induced X-chromosome inactivation, in addition to their putative roles in translational regulation.

Gaining insight into the scope and patterns of heterogeneity is a good starting point to improve our understanding of ribosome specialization. Recognizing consistencies and irregularities across different types of ribosome heterogeneity—such as the triggers for their formation, and the clustering and evolution of heterogeneous components—could help us group putative examples of specialization by their heterogeneous features, potentially filling the mechanistic gap between heterogeneity and specialization.

## 4. Mechanisms of translation regulation by specialized ribosomes

Categorizing a ribosome population as specialized requires an understanding of how its specific composition enables translational regulation. This means elucidating how the ribosome selects specific mRNAs to translate from the cellular pool of transcripts, or how it affects the translational levels of specific mRNAs or ORFs. To date, there are several different mechanisms by which specialized ribosomes have been found to function (e.g. initiation at start sites with slightly different Kozak sequences and mRNA selection via 5ʹ-untranslated region (5-UTR) elements) [6,11,18] that have been extensively reviewed [27,35]. These include the translation of ORFs with weak Kozak sequences by ribosomes lacking eS26. The loss of eS26 from ribosomes is induced during stress conditions by Tsr [36]. While mouse ribosomes containing eL38 preferentially translate HOX mRNAs, likely recruited by elements within 5′-UTRs [17]. Here we will provide an overview of these established mechanisms, and discuss additional putative mechanisms taking into account the fundamental characteristics of the ribosome and the wide variety of *cis*- and *trans*-acting regulatory components.

### (a) mRNA selectivity

One of the more established mechanisms by which specialization occurs is through the preferential recruitment to particular transcripts to enhance their translation. Several reports indicate that there are preferences for mRNAs without pointing to specific features in the mRNA through which ribosomes are recruited [13,32]. However, other examples suggest specific *cis*-acting elements in mRNAs directly influence the ribosome’s ability to bind elements in the mRNA, such as internal ribosome entry sites (IRESs) [37,38]. Such direct regulation has been observed in specific paediatric leukaemia, wher the RPL10 R98S mutation facilitates increased translation of BCL-2 via its IRES [39]. Additionally, regulatory mechanisms can be envisaged that could drive mRNA selectivity of specialized ribosomes through interactions with translation factors and non-coding RNAs.

### (b) Selection by upstream open reading frames

uORFs are short ORFs, located 5′ of the major protein coding ORF (mORF) in around half of all mRNAs in animals and plants. uORFs can be broadly divided into two categories [40,41]. By far the largest category of uORFs attenuate translation of their downstream mORF by interrupting ribosome scanning and thereby limiting ribosome access to the initiation codon of the downstream mORF. In general, these uORFs show little sequence conservation between species, and most, but not all, appear to calibrate the translation of their mORFs, rather than responsively regulating it [42]. A second, much rarer category of uORFs, accounting for only about 1% of uORFs in animals and plants, can play a more active regulatory role by providing a rapid, direct and flexible mechanism to control translation of their downstream mORF [43]. This class of uORF is characterized by amino acid sequence conservation, resulting in them being known as conserved peptide upstream ORFs (CPuORFs) or upstream conserved coding regions (uCCs) [44,45]. The peptide sequence of this uORF subset is essential to its ability to dynamically regulate translation of its mORF, in response to signals such as metabolite availability and stress conditions [46].

Examples of uORFs regulating mORF translation are found in different kingdoms of life, using a wide variety of modes of action. For example, in yeast, translation of the mORF encoding the GCN4 transcription factor, which allows yeast to adapt to starvation, is regulated by multiple uORFs, via a delayed reinitiation mechanism. In nutrient-rich conditions, the scanning ribosome translates the first uORF (uORF1) and quickly reinitiates translation, owing to high levels of the eIF2-GTP-Met-tRNAiMet ternary complex (TC). Consequently, the scanning ribosome goes on to translate another downstream uORF (uORF3 or uORF4), limiting the translation of the downstream GCN4 mORF. However, starvation conditions result in phosphorylation of eIF2, reducing TC levels. In this condition, after translating uORF1, reinitiation is delayed as a result of decreased availability of TC, allowing the scanning ribosome to pass the subsequent uORFs before reinitiation takes place at the GCN4 mORF [46]. A completely different mechanism is seen in the uORF-dependent regulation of the plant metabolic regulator bZIP11. In *Arabidopsis*, a uORF (uORF2) upstream of the mORF that encodes the bZIP11 transcription factor senses intracellular sucrose abundance, promoting ribosome stalling during translation termination under high sucrose concentrations and thus reducing translation of bZIP11 under those conditions [47]. In this case, structural analysis of the stalled ribosome suggests that a combination of exit tunnel ribosomal proteins and rRNA, together with the nascent uORF peptide, allows the translating ribosome itself to act as a metabolite sensor, to regulate translation of the mORF. The fact that analogous examples exist in other kingdoms suggests that uORF-mediated ribosome sensing of small molecules might be an evolutionarily conserved regulatory mechanism [48]. The wide range and flexibility of translational regulation mechanisms afforded by uORFs makes them attractive candidates for interaction with specialized ribosomes. Currently, the best example is the differential translation of specific KSHV uORFs and their mORFs, described above, which is influenced by rRNA modification [18,49]. However, it is likely that further work to understand both the mechanisms of translational control by uORFs and ribosome heterogeneity will reveal more examples of specialized ribosomes acting though one of the translational regulation mechanisms that depend on uORFs.

### (c) Selection by non-coding RNAs

In addition to direct protein interactions, ribosomes also interact with a group of non-coding RNAs (ncRNAs) termed ribosome-associated ncRNAs (rancRNAs). rancRNAs consist of a wide range of non-coding transcripts and have the capacity to influence translation, most often by general repression [50]. While the identification and verification of many rancRNAs is ongoing, to exclude any possibility that they are associated with ribosomes owing to their own translation, several rancRNAs have been shown to trigger global downregulation of translation.

The rancRNA_s194, in Archaea, inhibits the translation of a specific mRNA transcript via a mechanism that appears to rely on ribosome association and on complementarity between the rancRNA and the mRNA [51]. Similarly, long-noncoding RNAs (lncRNAs), which are not translated, have been found to be associated with ribosomes and could provide a means of specialization [52]. Owing to the abundance of potential for regulation by ncRNAs, if a ribosome population were able to recruit or exclude these regulatory molecules owing to the inclusion of different paralogues, rRNA sequences or modifications, this could provide an additional mechanism by which heterogeneous ribosomes could exert specialized translation over an mRNA pool.

### (d) Selection through specialized translation machinery

**Figure 3.**
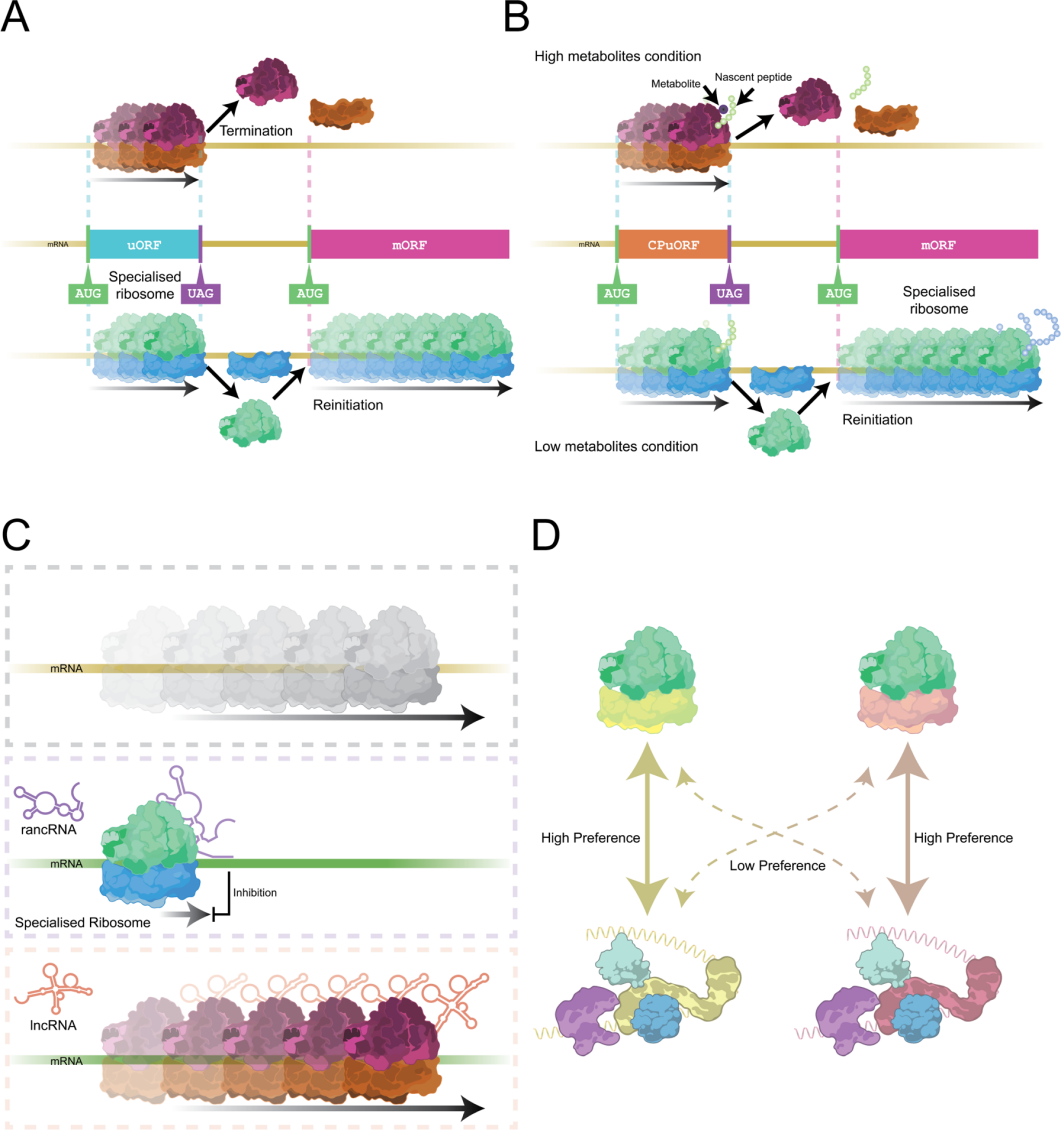
Additional mechanisms of translational regulation by specialized ribosomes. (A) Specialized ribosomes may be able to preferentially bypass or engage with regulatory upstream open reading frames (uORFs) to regulate translation of a downstream mORF (main ORF). (B) Heterogeneous ribosome populations could respond differently to the sensing of metabolites at conserved peptide upstream open reading frames (CPuORFs). (C) Certain non-coding RNAs (ncRNAs), including rancRNAs (ribosome-associated noncoding RNA) and lncRNAs (long-noncoding RNAs) can also associate with ribosomes and lead to specialized ribosomes regulating specific transcripts. (D) Subsets of mRNAs (yellow versus pink) might be bound by specialized initiation factors (yellow versus pink), which could act in association differentially with different populations for the selection of specific mRNAs.

For an mRNA to be translated by the ribosome, it is normally bound by several translation initiation factors first, which aid in recruiting the ribosome and prepare the transcript for start codon recognition [53]. In most eukaryotes, there are several paralogues and isoforms of translation initiation factors, with emerging data suggesting that these paralogous factors may have different affinities for subsets of mRNAs, influencing which are translated [54]. For example, multiple paralogues of eIF4G exist in all eukaryotes, many of which have been shown to interact with distinct mRNAs, leading to well defined translational consequences [55,56]. Specifically, eIF4G2 (DAP5) facilitates leaky scanning through uORFs and/or reinitiation at the main downstream coding sequence (CDS), contributing to ORF selection [57]. Additionally, eIF3 is a factor that can consist of essential and non-essential subunits that have also been shown to influence the translation of particular transcripts [58,59]. The idea of specialized translation machinery becomes increasingly complex when we consider the potential for heterogeneous initiation factor–mRNA complexes during the preparation of mRNA for interaction with the ribosome. While we have seen that both initiation factor and ribosomal protein heterogeneity can contribute to mRNA translation selectivity, whether these mechanisms are linked is unknown (figure 3). As it stands, we do not know exactly how different ribosomal proteins selectively interact with subsets of mRNAs, nor how different initiation factors confer a translational advantage to their bound transcripts. Indeed, the unknowns in both mechanisms could lend themselves to a theory of collaboration between different aspects of the heterogeneous translation machinery, one in which the definition of a ‘specialized ribosome’ is stretched by the extension of its mechanism to the role of other factors. While no examples of the aforementioned are currently known, additional experiments in characterizing ribosome heterogeneity, such as cross-linking mass spectroscopy (XL-MS), could help to elucidate if such a mechanism exists [56,60].

### (e) Regulation of translational elongation and co-translational folding

Previous reviews have suggested potential mechanisms of regulation mediated by specialized ribosomes during translation elongation [27] and recent work has provided a more detailed understanding of how this could occur. The methylation of eL40 by SMYD5 enhances translation elongation rates of specific mRNAs, contributing to the progression of gastric adenocarcinoma malignancy [20]. *eL39-like* (*eL39L*) is a mammalian-specific paralogue that exhibits tissue specific expression in testes, embryonic stem cells and cancers [61–63]. Depending on the cell type, multiple translational phenotypes have been reported from deletion of *eL39L* in mice: in mESCs, *eL39L* knockout reduces translation efficiency of Golgi- and endoplamic reticulum (ER)-component mRNAs; while in testes, differentially expressed proteins in *eL39L* knockout sperm do not exhibit changes in translation efficiency [11,64]. In general, both sets of targeted mRNAs preferentially translated by eL39L-containing ribosomes require co-translational folding of helices of the synthesized proteins [11,64]. However, different models have been proposed for the mechanism by which eL39L-containing ribosomes may facilitate co-translational folding (see below). Although peptide folding occurs during elongation, it could still result in the apparent selective translational regulation of specific proteins. Given eL39L is located at the nascent peptide exit tunnel (NPET), it seems unlikely that paralogue switching of eL39L could facilitate selective binding to specific transcripts. Rather, eL39L could enhance the stability of specific peptides during translation and co-translational folding. This fits with data showing that, in the absence of eL39L, markers of the unfolded protein response are elevated [11,64]. This may point to a mechanism in which neither eL39- nor eL39L-containing ribosomes exhibit a translational preference for specific mRNAs, but rather peptides that require co-translational folding are inefficiently translated by eL39-containing ribosomes, leading to increased misfolding stress response, degradation of the bound mRNA and subsequently the apparent decrease in translation efficiency.

## 5. Progress towards structural definitions of specialization mechanisms

While there is mounting evidence describing ribosome specialization, most of the examples of these different types of translation regulation by heterogeneous ribosome populations lack structural characterization that explains the underlying mechanisms of translational regulation. For example, loss of eS26, one of the best characterized examples of specialization, has been shown to regulate translation during stress in *S. cerevisiae*, by the use of start codons in poor Kozak sequence context [6]. Structural evidence for eS26’s interaction with the −4 position of the start codon in the P site supports the idea of eS26’s role in start codon selection [65]. However, a more in-depth mechanistic understanding would benefit from comparing structures with and without eS26, on mRNAs with different Kozak sequences.

Paralogue switching events provide a more challenging structural situation, because paralogues share a high degree of sequence similarity and require high-resolution structures to differentiate between them [3]. One such example is the paralogue pair uL3L and uL3 in mice, which share 78% amino acid identity. uL3L is specifically expressed in heart and skeletal muscle and regulates the translation of mRNAs involved in cardiac muscle contraction and dilated cardiomyopathy. The evolutionarily conserved differences between uL3 and uL3L sequences result in changes in amino acids near the A site [34]. These structural differences may induce a change in translation elongation dynamics and affect the tunnel entrance for the nascent peptide [34].

A more structurally justified mechanistic explanation has been provided for the tissue-specific involvement of eL39 and eL39-like paralogues in the translation of male germ-cell-specific proteins that are essential for the formation of sperm in mice (see above) [11]. High-resolution cryo-electron microscopy (cryo-EM) structures of ribosomes from mouse kidney and testis have revealed that eL39-like specific amino acid differences R28Q and R36M exhibit altered sidechain positions, when compared with eL39. This results in peptide exit channel expansion, facilitating the folding of nascent peptides into α-helices. Furthermore, the conserved positively charged R36 of eL39 rather hydrophobic M36 in eL39L paralog results in a modification of the surface properties of the tunnel, which has been proposed to regulate the folding of the proteins translated from the regulated mRNAs [11]. This hypothesis is further supported by cryo-EM of a chimeric complex of a yeast ribosome with the eL39L protein from *Mus musculus*, in which eL39 I35 and M36 present two alternative conformations that may aid the efficient co-translational folding of α-helices [64]. However, it should be noted that neither report showed direct interactions between the peptide exit tunnel and the nascent peptide in ribosomes containing the eL39L paralog. To structurally characterize the specific interactions between the nascent peptide and eL39L, and how these stabilize co-translational folding requires additional structural analysis, for example by cryo-EM or structural mass spectrometry of specific ribosomal populations trapped in the process of producing different types of nascent peptides.

A more complicated situation is observed in cases where tissue-specific paralogues of ribosomal proteins have flexible regions. For example, the most divergent region between eL22 and eL22-like in *D. melanogaster* is the N-terminal tail, which is localized on the exterior of the ribosome, near the peptide exit channel. It has been suggested that eL22 paralogues could be involved in ribosome specialization during spermatogenesis and their N-terminal tails could mediate this [66]. Similarly, in *D. melanogaster* the N-terminal tails are the most divergent regions between the uS7a and uS7b paralogues. uS7 is located on the head of the small ribosomal subunit, close to the decoding centre and contacting eIF2α, which modulates start codon recognition [67].

Neither uS7 nor eL22 paralogue N-terminal tails have been resolved in cryo-EM density owing to their flexibility [3]. In the cryo-EM structures from *D. melanogaster* the remaining amino acid residues of uS7A/uS7B paralogues form a tissue-specific surface on the head of the ribosome, directed away from the previously discovered uS7’s contact areas with eIF2 and mRNA channel in the 48S preinitiation complex from *S. cerevisiae*. The absence of complete structural data does not allow any direct conclusions regarding the structural and functional differences between these paralogues. Based on their specific location on the ribosome surface, one might hypothesize that these flexible N-terminal tails could form a specific surface (eL22/eL22-like) or expand existing surface (uS7A/uS7B) for the association of different factors on the ribosome and thereby contribute to ribosome specialization. Future experiments to understand differences in binding partners, using other structural approaches to complement EM, could help to identify any contribution to specialization by paralogue-differing N-termini.

## 6. Methods to define specialization and their limitations

Work over recent years to characterize specialized ribosomes has utilized complementary functional and structural studies. While structural studies provide great insight to the mechanistic basis of specialization, functional work has focused on disrupting ribosome components and characterizing resulting phenotypes.

### (a) What experiments are required to show specialization?

Although structural knowledge provides the gold standard for the basis of mechanistic insight many studies have focused on disrupting ribosome components and characterizing resulting phenotypes. A common critique of the specialized ribosome theory is that loss-of-function experiments (or other experimentally introduced perturbations) can cause a reduction in global translation levels. This will likely generate a phenotype that could masquerade as specialization. To assess this, ribosome subunit abundance can be determined by sucrose gradient fractionation, identifying the relative abundances of subunits and the total quantity of ribosomes [68]. Modern genetic experiments can minimize genetic disturbance and therefore reduce the likelihood of off-target effects or global translational dysregulation. With CRISPR-based approaches, a small tag (such as FLAG or HA) can be introduced to a specific ribosomal protein paralogue in its native context [33]. This may allow the isolation of specific pools of ribosomes without altering the amount of total ribosomal machinery or causing off-target effects. Studying ribosomes *in situ* will also likely reduce experimentally introduced artefacts and better retain transiently bound cofactors [69]. Focused ion beam (FIB)-milling and cryo-electron tomography (cryoET) have both benefitted from recent advancements in hardware, data collection and image processing and may yield key mechanistic insights by minimally perturbing ribosomes [69].

### (b) Demonstrate which function is responsible for a phenotype

One complication in characterizing specialization is the dual functions played by many ribosomal-associated proteins. Many ribosome biogenesis factors have multiple functions, for example BUD23 and EMG1 are both assembly factors and methyltransferases [70,71]. The essential function of EMG1 may be as a chaperone to aid incorporation of eS19 during biogenesis; therefore a phenotype resulting from loss of EMG1 may be the result of an eS19 deficiency, rather than a regulatory function of the ribosome [70,72].

Knockdown of both fibrillarin and dyskerin rRNA modification enzymes has been shown to result in reduced IRES-mediated translation events [73,74]. Interpretation of these results is challenging as the knockdown of these enzymes can perturb ribosome biogenesis and evoke stress response, for example the activation of p53 by fibrillarin knockdown [75]. A more fine-tuned approach is needed to explore the importance of rRNA modifications to ribosome function, such as knocking down individual small nucleolar RNAs (snoRNAs) [13,76].

### (c) Demonstrate a regulatory capacity, not just a disease or stress phenotype

Some types of heterogeneity result in defective ribosomes rather than constructive regulation of translation [77,78]. It has been argued that this is aberrancy, not specialization [79]. Defective ribosomes can stall, leading to ribosomal collisions, subunit disassembly and initiation of the integrated stress response [80]. It has been suggested that ribosome collisions could be a method of purifying heterogeneity from a ribosome pool [79]; just because a ribosome has a particular composition and is assembled on a transcript does not necessarily mean that it is performing a function. General stress response to such changes should be assessed and, if known stress responsive genes show an altered expression profile between control and experimental conditions, a general translational deficiency should be considered. Visible symptoms of translational deficiency could also be observed, such as poor cellular growth in tissue culture-derived cells, or retarded growth in whole organisms [81,82]. Di-somes (containing collided ribosomes) can also be sequenced, much like the typical ribosome profiling pipeline [83,84].

### (d) Demonstrate specialization using an appropriate model

Cell culture is commonly employed to imitate biological systems. However, this convenience could be hampering discovery, as rapidly proliferating tissue culture-grown cells may place an emphasis on bulk ribosome synthesis for large amounts of new proteins, minimizing the production of highly specialized ribosomes [79]. High translation levels can be observed in ribosome profiles from cell lines, for example embryonically derived cultured *D. melanogaster* S2 cells have vastly more polysomes than embryos harvested *in vivo* [3]. Techniques such as ribosome profiling can be implemented with smaller input material than previously and can be performed gel-free [85], making high-level experiments on *in vivo* tissue more feasible [86]. Even cryo-EM has been combined with immuno-precipitation (MagIC-cryo-EM), to generate structures from low concentrations of heterogeneous samples [87].

## 7. Using viral systems to characterize specialized ribosomes

The selection of biological models will continue to be important within the specialized ribosome field to ensure we characterize genuine examples. Viruses lack their own translational machinery and therefore they rely exclusively on the host cell ribosomes for the synthesis of viral proteins. As such, viruses have evolved diverse mechanisms to ensure translational efficiency of viral mRNA goes above and beyond that of cellular mRNA. These processes serve to redirect the translation apparatus to favour viral transcripts. Emerging evidence suggests that viruses can manipulate the production of specialized ribosome populations, creating virus-specific specialized ribosomes to enhance the translation of their own viral mRNAs [18,88]. Virally induced specialized ribosomes have the potential to highlight highly conserved, endogenous mechanisms of specialization to explore further in other systems. For example, RACK1 is not essential for global translation but instead is required for efficient translation initiation of mRNAs with short ORFs that show greater than average translational efficiency in eukaryotes [89]. Interestingly, RACK1 is also a target for poxviruses, which specifically phosphorylate serine/threonine residues within the extended loop of RACK1, resulting in ribosome selectivity towards post-replicative viral RNAs with 5′-UTR poly A-leaders [90,91]. In yeast, eL40-dependent translation initiation of vesicular stomatitis virus (VSV) transcripts has been also shown to be conserved among a subset of cellular mRNAs upregulated during cellular stress [92]. This suggests that poxviruses and VSV may utilize endogenous mechanisms of ribosome specialization to enhance viral protein synthesis. Moreover, eL40 specialization may be specific to the cellular stress response. Virally infected cells can provide an endogenous representation of cellular stress, mitigating the need for more artificial forms of stress induction.

### (a) Mechanistic insight into internal ribosome entry site utilization by viruses

IRES elements are structured RNA sequences within the 5′-UTR of viral and cellular mRNAs that allow translation initiation in the absence of a canonical eukaryotic m^7^G cap [93]. Recent evidence has suggested specialized ribosomes may play a role in the cap-independent translation of viral transcripts. Multiple studies have shown ribosomal proteins eS25 and uL1 directly contact viral IRES elements of Cricket paralysis virus (CrPV) and HCV mRNAs and are essential for their translation [4,94,95]. Despite this, the role of specific ribosomal proteins in cellular IRES-mediated translation is poorly understood. Interestingly, one study identified several cellular transcripts that preferentially associate with uL1-containing ribosomes and have uL1-dependent IRES activity, and eS25 has also been previously shown to regulate cellular IRES activity [96,97]. Given the potential for hundreds of IRES elements being present in the human genome, characterizing viral IRES-mediated translation initiation could elucidate mechanisms of ribosome specialization, which may also be driving cellular cap-independent translation, reflecting a common regulatory strategy [97].

### (b) Uncovering dual functionality of associated factors in viral models

Ribosome biogenesis is an inherently complex process, dynamically responding to the changing cellular environment, development and disease [98]. Higher eukaryotic cells encode over 400 ribosome biogenesis factors which modulate ribosome composition and have been shown to fine-tune the functional activity of the ribosome. This can make identifying highly specific pathways leading to the production of heterogeneous ribosome populations challenging. Therefore, characterization of specialized ribosomes in virally infected cells offers a binary system in which changes in ribosome biogenesis can be objectively measured and analysed under controlled conditions. For example, the ribosome biogenesis factor BUD23 was shown to have enhanced association to the pre-40S ribosomal complex during KSHV lytic replication compared with latent infection [18]. Further analysis has highlighted that the methyltransferase activity of BUD23 and increased methylation of the 18S rRNA base G1639 are critical for the efficient translation of late lytic structural genes and subsequent infectious virion production. Specifically, increased association of BUD23 reduced the translation of specific uORFs present in KSHV late lytic transcripts, which in turn enhanced the translation of the downstream CDSs. Interestingly BUD23 and m^7^G1639 have been implicated in the preferential translation of cellular mRNAs with low 5′-UTR GC content [49]. As BUD23 impacts the translation of mRNAs with low 5′-UTR GC content and/or certain uORF types this could help explain the variety of symptoms observed in the genetic disease Williams syndrome, in which BUD23 and other genes are deleted [49]. Therefore, utilizing viral systems can help to identify often intricate changes in the highly complex process of ribosome biogenesis, illuminating the pathways by which specialized ribosomes may be produced.

## 8. Future challenges

New technical advances will be required to understand the true extent of ribosome heterogeneity and distil the detailed mechanisms of specialization *in vivo*, particularly as specific ribosome populations may represent only a subset in a small cell population. For example, high-resolution single particle cryo-EM is beginning to help determine the impact of rRNA modifications, thus providing important mechanistic insight [99]. In addition, time-resolved cryo-EM [69], atomic force microscopy (AFM) [100] and single-molecule fluorescence resonance energy transfer (smFRET) are currently used to capture the dynamics of translocation and ribosome kinetics [101], which will likely be important as we dissect the impact of modifications to the ribosome on elongation. Understanding how changes in ribosome composition affect ribosome structure and movement is an important step in unravelling precisely which function is causing a phenotype. Direct RNA sequencing (dRNA-seq) is beginning to accurately and robustly map rRNA modifications, identifying modifications that co-occur on the same ribosome [102]. This will be useful in untangling the effect of multiple changes to ribosome composition, by combining specific RP immunoprecipitations (IPs) with nanopore sequencing [33]. This would go towards addressing an outstanding question to determine whether patterns in terms of combinations of heterogeneity exist. For example, do some compositional changes result in additional compositional changes nearby or elsewhere on the ribosome (e.g. loss of 18S rRNA U3904 methylation leads to fragile X mental retardation protein (FMRP) association) [5].

dRNA-seq relies upon monitoring changes in ion current across a nanoscale pore, which occur during the passage of a single nucleic acid molecule through the nanopore [103]. However, despite the exponential growth of this technique, challenges still do exist, and other methods (e.g. mass spectrometry, NMR, cryo-EM) are often required to confirm de novo identified modification sites through nanopore dRNA-seq. The current bioinformatics tools for RNA modification mapping from dRNA-seq data [104] still have limitations: (i) there is modest overlap among different tools, with plenty of tool-specific identified sites that might be considered false-positive; (ii) training models tend to be organism-specific, owing to the use of cell line mutants knocked out for specific modifying enzymes as the unmodified controls; and (iii) in cases where tools do model training on the user-provided dataset, wet lab experiments are required to produce knockout mutants and/or *in vitro*-transcribed rRNAs.

Nanopore technology can also be employed to structurally fingerprint intact biomolecules, and it has been used in recent years to analyse ribosome samples with single-entity resolution. Nanopores allow ribosomal analysis in very small volume (3–5 μl), requiring minimal sample preparation, and can provide quantitative results within minutes. Nanopore–optofluidic devices have been developed for the controlled translocation and counting of 70S ribosomes from a DNA–ribosome mixture [105]. Further still, glass nanopores have successfully been used to fingerprint 80S ribosomes and polysomes from a human neuronal cell line and *D. melanogaster* cultured S2 cells and ovaries. The ion current signals of 80S ribosomes are distinct from polysomes and can be used to discriminate ribosomes from polysomes in mixed samples and demonstrate a correlation between polysome size and signal amplitude [106]. This nanopore approach did not have the resolution to distinguish monosomes from small polysomes, but recent advances in the detection sensitivity of glass nanopores hold great potential for improved discrimination of different ribosome complexes [107,108].

Other exciting developments around nanopore technologies lie in the integration of fluorescence readout, allowing the combination of the large temporal bandwidth of nanopore sensing with the sub-nanometre resolution of fluorescence techniques like single-molecule FRET (single-molecule FRET having been used in the past to complement cryo-EM to reveal the dynamics of ribosome function and to study ribosomal translocation dynamics) [109–111]. These improvements could make use of nanopores an alternative to sucrose density gradient ultracentrifugation, complementing the cryo-EM and mass spectrometry toolbox for the structural analysis of different ribosome populations.

## 9. Final remarks

Historically, the theory of specialized ribosomes has been based on the ribosome filter hypothesis, in which different ribosome populations regulate particular sets of mRNAs [112]. Generally, there has been a focus on the ability of different ribosomes to preferentially translate subsets of mRNAs [113]. There are numerous examples of such specialization, but it is important to emphasize that specialized ribosomes are not simply selecting different mRNAs to translate. By deepening our understanding of specific examples, numerous mechanisms of regulation have been revealed (figure 3). As we have described, there are many ways in which heterogeneous ribosomes could and do regulate translation. A more inclusive and accurate definition of ribosome specialization is that differences in ribosome composition affect the ability to regulate translation of specific mRNAs or ORFs. If a ribosome is deficient, potentially from lacking a RP, and cannot therefore perform translation, this would not be a specialized ribosome [36]. We would like to put a greater emphasis on the ORF and resulting peptide in the impact of heterogeneity. There are well characterized examples where changes in ribosome composition impact its ability to select a start codon and therefore select an ORF. This includes RpS26 in yeast and changes in uORF translation levels upon rRNA methylation [6,18]. Although changes in the association of ribosome-bound factors can regulate translation, we would only consider this ribosome specialization if the ribosome’s activity were being affected, rather than an RNA-binding factor affecting 40S recruitment. A more subtle scenario to consider is that a change in ribosome composition may impact the association of factors with the ribosome. For us, another key aspect of defining specialization in ribosomes is understanding how such changes in ribosome activity have evolved. This will require the field to do more extensive comparisons across multiple species and systems to understand when and how specialization evolved and how widespread it is.

## Data Availability

This article has no additional data.
